# The Relationship Between Fatigue and Quality of Life in the Turkish Mothers of Children with Autism Spectrum Disorder

**DOI:** 10.1007/s10803-024-06398-z

**Published:** 2024-05-20

**Authors:** Koray Kara, Ozgun Kaya Kara, Gulsah Sutcu, Hasan Atacan Tonak

**Affiliations:** 1 Department of Child and Adolescent Psychiatry, University of Health Sciences, Antalya Training and Research Hospital, Antalya, Turkey; 2 Faculty of Health Sciences, Department of Physiotherapy and Rehabilitation, Akdeniz University, Antalya, Turkey; 3 Hamidiye Faculty of Health Sciences, Department of Occupational Therapy, University of Health Sciences, Istanbul, Turkey

**Keywords:** Autism spectrum disorder, Fatigue, Quality of life, Function, Health, Mothers

## Abstract

This study aimed to investigate the different effects of fatigue and health-related quality of life in the Turkish mothers of children with autism spectrum disorder (ASD) through comparisons with mothers of typically developing children, and to evaluate the relationship between different aspects of maternal fatigue, depression, and maternal health-related quality of life. The study included a total of 103 mothers, comprising 60 mothers of children with ASD and 43 mothers of typically developing children. The Nottingham Health Profile and Quality of Life in Autism Questionnaire-Parent Version were used to assess the health-related quality of life. Fatigue was assessed comprehensively with the Fatigue Impact Scale and the Fatigue Severity Scale. The Nottingham Health Profile total and physical mobility domain and the quality of life were the strongest factors, explaining with a variance of 66.7% the fatigue impact on cognitive function. The mothers’ quality of life accounted for 64.8% of the variance in factors that explained fatigue. Physical mobility, social isolation and quality of life were associated with the physical impact of fatigue and were explained with a variance of 52.4%. Emotional reactions and quality of life were explained with a variance of 52.7% in the fatigue of psychosocial function. The current study has highlighted that the fatigue of Turkish mothers of children with ASD has a greater impact on cognitive, physical and psychosocial functions. Furthermore, the quality of life, physical mobility, social isolation, and emotional reactions of mothers have a significant impact on maternal fatigue.

Autism spectrum disorder (ASD) is one of the most common lifelong neurodevelopmental disorders, characterized by persistent social communication and interaction difficulties, stereotypical behaviors, and restricted interests (Association, American Psychiatric Association [APA] 2013). The incidence of ASD has increased considerably during the past decade (Chiarotti & Venerosi, 2020). Recent meta-analyses have estimated the global prevalence of ASD to be 72 per 10,000 population (Talantseva et al., 2023), and a prevalence study conducted in Turkey of children aged 16–36 months revealed ASD incidence to be 1 in every 117 children (Oner & Munir, 2020). ASD has become a worldwide health issue that places substantial financial and health burdens on families as well as the community (Jariwala-Parikh et al., 2019; Talantseva et al., 2023).

Families of children with ASD have numerous challenges such as providing daily care, managing medical treatments, and addressing the child’s behavioral problems (Turnage & Conner, 2022). In addition to dealing with the main symptoms of ASD, parents have to deal with the emotional and behavioral challenges that arise from constantly supervising and caring for their child (Tsai et al., 2020). This explains why parents of children with ASD report much higher levels of stress compared to parents of typically developing children and those with other developmental problems (Schnabel et al., 2020). The negative impact of stress on the quality of life (QoL) of parents with ASD progressively worsens over time (Turnage & Conner, 2022).

The World Health Organization (WHO) defines QoL as “an individual’s perception of their position in life in the context of the culture and values systems in which they live and in relation to their goals, expectations, standards and concerns”(The World Health Organization Quality of Life assessment (WHOQOL): position paper from the World Health Organization, 1995). Fatigue, which is a crucial component of QoL and wellbeing, is the feeling of physical and mental burnout that cannot be overcome with rest (Giallo et al., 2013; Seymour et al., 2013). In addition, serious health issues, such as difficulty with attention, memory, decision-making, and coping with children, are caused by fatigue in mothers of typically developing children (Nyberg & Sternhufvud, 2000). The well-being of a mother has an impact not only on themselves, but also on the development of their children (Seymour et al., 2013). Unfortunately, clinical practice and research often neglect the well-being of mothers raising children with ASD (Seymour et al., 2013). Therefore, there is a critical need for studies that specifically examine the well-being of mothers of children with ASD.

Raising a child with ASD can be more challenging for parents in middle and low-income countries than in the rest of the world, resulting in emotional exhaustion, a decreased QoL, financial difficulties, and feelings of helplessness (Daniels et al., 2017tük et al., 2021; Özgür et al., 2018; Suen et al., 2021). The majority of research on the well-being of parents of children with ASD has been performed in high-income countries, with the largest number of such studies in Europe, whereas findings from low and middle income countries are limited (Scherer et al., 2019). Therefore, in the light of the worldwide focus on parents of children with ASD, it is crucial to evaluate the well-being of parents of children with ASD in developing countries as well as in developed countries (Ağırkan et al., 2023). Due to its strategic geographical location, Turkey is a bridge between the western and eastern regions of the world. Throughout the centuries, it has been subject to the impact of various cultures, including Western, Asian, Mediterranean, and Islamic. Culture is significantly influenced by behaviors, attitudes, and how parents react towards their children with disabilities (Kolhatkar & Berkowitz, 2014; Susanty et al., 2021). In traditional Turkish culture, parents believe that the reputation of their family is directly related to their child’s achievement. Thus, when a child with a disability fails to perform up to expectations, parents usually feel a great deal of guilt (Sage & Jegatheesan, 2010). Then, Turkish parents of a child with a disability may frequently refuse to discuss their child (Ağırkan et al., 2023). The Turkish family structure maintains a hierarchical system based on gender and generations This also affects parenting responsibilities, with women still responsible for providing the primary care of children, regardless of their education level or employment status (Turkdogan et al., 2019). This may result in an increased emotional burden, fatigue, and diminished quality life. Hence, the outcomes obtained from population-based samples in Turkey can contribute to the existing body of knowledge (Saral et al., 2023).

In recent years, there has been an increase in the number of studies conducted in Turkey on the parents of children ASD that have investigated the factors affecting QoL, anxiety, depression and burden (Baykal et al., 2019; Kuru & Piyal, 2018ük et al., 2021; Özgür et al., 2018; Tekinarslan, 2013; Yıldız et al., 2016). However, none of the studies have examined the relationship between fatigue and the QoL of mothers of children with ASD through the use of multiple linear regression analysis and a specialized questionnaire designed to assess the QoL of mothers of children with ASD. There are a few studies that have investigated the fatigue of mothers of children with ASD and have reported that they experienced greater levels of exhaustion, physical and mental fatigue, which increased in line with the behavioural difficulties of the child compared to the mothers of typically developing children (Giallo et al., 2013; Seymour et al., 2013; Smith et al., 2010). Although these studies indicated that it is absolutely necessary to assess the levels of fatigue experienced by mothers of children with ASD (Giallo et al., 2013; Seymour et al., 2013; Smith et al., 2010), there has been no focus on the different effects of fatigue on mental, physical and psychosocial functions in the Turkish population.

Therefore, the aim of this study was to investigate (1) the different effects of fatigue and health-related QoL in Turkish mothers of children with ASD through comparisons with the Turkish mothers of typically developing children, and (2) the relationship between different aspects of maternal fatigue, depression, and maternal health-related QoL using a multifactorial method. The study hypotheses were that.

1. mothers of children with ASD have greater mental, physical, and psychosocial effects of fatigue and lower health-related QoL than mothers of typically developing children.
2. high levels of maternal fatigue are substantially associated with lower levels of health-related QoL, and increased depressive symptoms.

## Methods

### Participants

The study included a total of 103 mothers, comprising 60 mothers of children and adolescents with ASD and 43 mothers of children and adolescents without ASD. Children and adolescents were dignosed with ASD by a child and adolescent psychiatrist, according to the criteria outlined in the fifth edition of the Diagnostic and Statistical Manual of Mental Disorders (DSM-V) (Association, American Psychiatric Association [APA] 2013), which include impaired social interaction and communication difficulties, abnormal and repetitive behavior, restricted interests and activities, and early childhood symptoms (Association, American Psychiatric Association [APA] 2013). The inclusion criteria for the mothers were as follows: (1) Mothers of children and adolescents with ASD aged 2–18 years, (2) mothers of typically developing children and adolescents aged 2 to 18 years, and (3) mothers who agreed to participate in the study. The exclusion criteria for the mothers were as follows: (1) mothers of children and adolescents with comorbid neurological disorders, such as cerebral palsy, epilepsy, syndromic or chromosomal disorders, (2) mothers who had been diagnosed with a psychiatric or chronic disorder, such as psychotic symptoms, diabetes mellitus, stroke, etc., based on their medical records.

### Procedure

Approval for this study was obtained from the University Local Ethics Committee. The mothers of all the study participants provided written consent in accordance with the principles stated in the Helsinki Declaration. Mothers who agreed to take part in the study completed the socio-demographic form and Nottingham Health Profile (NHP), Fatigue Impact Scale (FIS) and the Fatigue Severity Scale (FSS), The Beck Depression Inventory (BDI) and The Quality of Life in Autism Questionnaire-Parent Version (QoLA). The children and adolescents with ASD included in the study were those who had been routinely referred to the child and adolescent psychiatry department. Children and adolescents without ASD were reached using the snowball sampling method. The clinical trial registration number is NCT05422261.

### Measures

#### Sociodemographic Questionnaire

All the mothers completed the questionnaire to provide information about the family’s cultural and socio-economic status, such as family income, maternal education level, employment status, family living place, and demographic data of mothers’ age and children or adolescents’ age and sex.

#### Nottingham Health Profile

The Nottingham Health Profile (NHP) was used to assess the health-related QoL of the mothers in the study. The NHP consists of 38 items in 6 subsections of pain (8 items), emotional reactions (9 items), social isolation (5 items), sleep (5 items), physical abilities (8 items) and energy level (3 items), and each item is answered yes/no. The total score ranges from 0 to 600 and each subsection can be scored within itself in the range of 0-100. High scores indicate a low QoL related to perceived health status (Hunt et al., 1981). The Turkish adaption study of the NHP found that the scale was valid and reliable, with test-retest reliability of subscales ranging from 0.70 to 0.92 and internal consistency scores ranging from 0.56 to 0.87 (Kucukdeveci et al., 2000).

#### Fatigue Assessment

Fatigue was assessed comprehensively with the Fatigue Impact Scale (FIS) and the Fatigue Severity Scale (FSS). The FIS consists of a total of 40 items in subsections of the cognitive (10 items), physical (10 items) and psychosocial (20 items) effects of fatigue. Each item is scored between 0 and 4. Each section is scored separately and the total score ranges from 0 to 160. Higher scores indicate greater effects of fatigue (Fisk et al., 1994). The Turkish version of the scale has been shown to have confirmed convergent and discriminant validity, as well as internal reliability (Cronbach’s alpha = 0.96) (Armutlu et al., 2007). The FSS, assessing the severity of fatigue, consists of 9 items and the individual is asked to select a number from 1 (strong disagreement) to 7 (strong agreement) indicating the level of agreement with each statement. The total score ranges from 9 to 63, and higher scores indicate increased severity of fatigue (Krupp et al., 1989). The Turkish version of the scale has been shown to have convergent and discriminant validity, as well as internal reliability of 0.89 (Cronbach’s alpha) (Armutlu et al., 2007).

#### Beck Depression Inventory

The Beck Depression Inventory (BDI) consisting of 21 items was used to assess behaviors and feelings related to general depressed mood. Each item is scored between 0 and 3 and the total score ranges between 0 and 63. Higher scores indicate higher levels of depression risk (Beck et al., 1987). The Turkish version of the BDI has been shown to be valid and reliable (Cronbach’s alpha = 0.89) (Hisli, 1988).

#### Quality of Life in Autism Questionnaire-Parent Version

The Quality of Life in Autism Questionnaire-Parent Version (QoLA) consists of two parts, A and B. Part A contains 28 items measuring how parents perceive their own QoL, and each item is scored on a five-point Likert scale ranging from one (not very much) to five (very much). Part B assesses parents’ perception of how much of a problem their child’s autism-specific difficulty is for them. This part contains 20 items about the difficulties experienced by children, and each item is scored on a five-point Likert scale from one (very much a problem for me) to five (not much of a problem for me). A higher score shows that parents experience fewer challenges as a result of their child’s ASD-related behaviors. While each part can be scored separately, the total score that can be obtained from the scale ranges from 48 to 240. Higher scores indicate that parents have a high QoL (Eapen et al., 2014). The Turkish version of the QoLA has been shown to have strong validity and reliability in terms of its psychometric properties (Özgür et al., 2017) .

### Data Analysis

Data obtained in the study were analyzed statistically using SPSS vn. 25.0 software (IBM, New York, USA). The variables were investigated using histograms and the Shapiro-Wilk test to determine conformity to normal distribution. Descriptive statistics for ordinal data were reported as mean and standard deviation values, and categorical variables as number and percentage. Continuous variables (age) were compared using the Student’s t test, and categorical variables (gender, marital status, education level, employment, family income, living place, number of children) with the χ² test. The Independent Samples t-test was used to analyze the difference in fatigue impact score, fatigue severity level, BDI and NHP scores between mothers of children with ASD and mothers of typically developing children. Multiple regression models were used to determine the associations between the fatigue domains, BDI score, and maternal QoL domains. Effect sizes (Cohen’s d) were calculated with GPower V3.1.7 (University of Kiel, Kiel, Germany) using the mean and standard deviation of the change in scores within the groups. Effect size was classified as large if > 0.8, moderate if 0.5 to 0.8 and small if < 0.5. A value of *p* < 0.05 was accepted as the level of statistical significance.

All the explanatory variables were included in the models with the stepwise method. Variance index factors, correlation tests, normal probability plots, scatter plots of the residuals, and histograms were used to test the normality and collinearity assumptions of multiple regression models. The Cook’s distance test was performed to detect skewed data. To interpret the results of regression analysis, standardized coefficient ß, 95% confidence intervals and adjusted R^2^ were used. Five models were found to be statistically significant (*p* < 0.05). According to the post-hoc power analysis performed using G*Power 3.1.9.4 (Universtat Düsseldorf, Germany), the study reached 96.4% power for FIS-cognitive × QoL domains of the regression model (α = 0.05, effect size = 2, non-centrality parameter λ = 28.04).

## Results

### Demographic Data

A total of 103 participants (n_mothers of children with ASD_ =60, n_mothers of typically developing children_ =43) were included in this study. The mothers of children with ASD and those of typically developing children were seen to have similar sociodemographic characteristics (Table 1). The average age of mothers was 37.8 years (SD = 5.8, range = 24–50), and the average age of the children was 9.13 years (SD = 4.03, range = 2-17.5). Most of the participants were married (88.3%), had moderate to high family income (86.4%), and lived in an urban area (85.4%). More than half of the mothers had a college or bachelor’s degree (68.8%) and had a son (63.1%). Less than half of the mothers (38.8%) were not employed outside the home and had only one child (32%).

**Table 1:** Demographic and clinical data of children and their mothers

Demographic data of mothers		Autism group (*n* = 60) mean±SD (min-max), *n* (%)	Control group (*n* = 43) mean±SD (min-max), *n* (%)	*p* ^a, b^
Age (years)		38.52±5.79 (24–50)	36.86±5.90 (28–48)	0.159^a^
Marital status	Married	51 (85)	40 (93)	0.351^b^
	Divorced	9 (15)	3 (7)	
Education level	Primary school	6 (10)	9 (20.9)	0.242^b^
	Secondary school	8 (13.3)	9 (20.9)	
	High School	18 (30)	10 (23.3)	
	University	28 (46.7)	15 (34.9)	
Employment		19 (31.7)	21 (48.8)	0.102^b^
Income	Low	10 (16.7)	7 (16.3)	0.194^b^
	Moderate	22 (36.7)	9 (20.9)	
	High	28 (46.7)	27 (62.8)	
Living place	City	54 (90)	34 (79.1)	0.211^b^
	Town	4 (6.7)	4 (9.3)	
	Village	2 (3.3)	5 (11.6)	
Number of children	One	22 (36.7)	11 (25.6)	0.152^b^
	Two	29 (48.3)	19 (44.2)	
	Three or more	9 (15)	13 (30.2)	
QoLA score (min-max)	A	97.02±21.55 (37–136)		
	B	67.65±18.51 (39–139)		
	Total	164.67±35.11 (82–247)		
Demographic and clinical data of children				
**Gender**	Female	18 (30)	20 (46.5)	0.101^a^
	Male	42 (70)	23 (53.5)	
**Age (years)**		8.64±3.93 (2.00-17.50)	9.82±4.12 (2.00–17.00)	0.144^b^

*p* < 0.05, ^a^Independent-Samples T, ^b^χ^2^ Test, QoLA: Autism Quality of Life

### Differences in Fatigue, Depression, and Health-Related Quality of Life Between the Mothers of Children and Adolescents with and without ASD

The differences in cognitive, physical, and psychosocial effects of fatigue, fatigue severity, depression level, and health-related quality of life between the mothers of children with ASD and typically developing children are shown in Table 2. The mothers of children with ASD had significantly higher scores in the Fatigue Impact Scale total and subdomain scores (cognitive, physical, psychosocial), Beck Depression Inventory scores, and Nottingham Health Profile total and subdomain scores (pain, emotional reactions, social isolation, sleep, physical mobility, energy level) with small to large effect sizes (0.40–1.07).

**Table 2:** Comparison of the scores of the mothers

		Mothers of children with autism (*n* = 60) mean±SD (min-max)	Control group (*n* = 43) mean±SD (min-max)	*p*	Mean Difference	Standard Error Difference	Cohens d
**FIS-cognitive**		10.27±9.52 (0–38)	4.79±5.16 (0–17)	< 0.001*	5.47	1.46	0.68
**FIS-physical**		8.32±7.71 (0–32)	3.98±3.85 (0–13)	< 0.001*	4.34	1.15	0.67
**FIS-psychosocial**		18.75±15.20 (0–53)	6.09±7.29 (0–31)	< 0.001*	12.65	2.25	1.01
**FIS-Total**		37.32±29.02 (0-120)	14.84±12.92 (0–45)	< 0.001*	22.47	4.23	0.94
**FSS**		37.37±13.17 (9–58)	33.47±11.09 (9–54)	0.117	3.90	2.46	0.31
**BDI**		12.13±7.58 (0–33)	5.40±3.75 (0–14)	< 0.001*	6.73	1.13	1.07
**NHP**	Pain	14.41±26.61 (0-100)	5.46±13.06 (0-62.47)	0.027*	8.95	3.97	0.40
	Emotional reactions	27.29±27.73 (0-100)	9.79±11.76 (0-42.98)	< 0.001*	17.49	4.00	0.77
	Social isolation	26.10±34.73 (0-100)	6.79±16.65 (0-55.46)	< 0.001*	19.31	5.15	0.67
	Sleep	32.60±27.40 (0-100)	17.88±19.46 (0-77.63)	0.002*	14.72	4.61	0.60
	Physical mobility	13.29±14.84 (0-54.83)	6.98±7.97 (0-21.77)	0.007*	6.30	2.26	0.50
	Energy level	35.54±37.30 (0-100)	21.46±27.08 (0-100)	0.029*	14.07	6.34	0.42
	Total	149.40±128.06 (0-444.79)	70.04±61.38 (0-200.93)	< 0.001*	79.35	18.99	0.75

*p < 0.05, Independent-Samples T Test, NHP: Nottingham Health Profile, BDI: Beck Depression Inventory, FSS: Fatigue Severity Scale, FIS: Fatigue Impact Scale

### Relationships Between Maternal Fatigue and Mother’s Quality of Life

The results of the multiple linear regression analysis are shown in Table 3. The NHP total and physical mobility domain and the mothers’ QoL related to having a child with autism were the strongest factors, explaining with a variance of 66.7% the fatigue impact on cognitive function. Based on beta coefficients, a mother’s poor QoL related to having a child with autism and lower level of physical mobility were associated with a higher level of fatigue that had an impact on cognitive functions. The mothers’ QoL related to having a child with autism accounted for 64.8% of the variance in factors that explained fatigue. Physical mobility, social isolation and QoL were associated with the physical impact of fatigue and were explained with a variance of 52.4%. Emotional reactions and the mothers’ QoL related to having a child with autism were explained with a variance of 52.7% in the fatigue of psychosocial function. When examining fatigue severity, physical mobility and QoL related to having a child with autism were explained with a variance of 29.8% in this domain.

**Table 3:** Stepwise-multiple linear regression analyses

Fatigue Domains	β Coefficient	95% Confidence Interval	*p*	*R*^2^ (%)
**FIS-cognitive**				
NHP-Total	0.028	0.008–0.049	0.007	66.7
NHP- Physical mobility	0.195	0.077–0.313	0.002	
Autism Quality of Life- Part A	−0.128	−0.23- -0.02	0.018	
**FIS-physical**				
NHP- Physical mobility	0.156	0.05–0.25	0.003	52.4
NHP-Social isolation	−0.062	−0.114- -0.01	0.021	
Autism Quality of Life- Part A	−0.262	−0.34- −0.17	< 0.001	
**FIS-psychosocial**				
NHP- Emotional reactions	0.18	0.03–0.32	0.001	52.7
Autism Quality of Life- Part A	−0.32	−0.50- −0.13	0.014	
FIS-Total				
NHP- Total	0.094	0.03–0.14	0.001	64.8
Autism Quality of Life- Part A	−0.603	−0.92- -0.27	< 0.001	
FSS				
NHP- Physical mobility	0.22	0.02–0.43	0.03	29.8
Autism Quality of Life- Part A	−0.26	−0.40- -0.11	< 0.001	

*NHP*: Nottingham Health Profile, *FSS*: Fatigue Severity Scale, *FIS*: Fatigue Impact Scale

## Discussion

This study compared the effect of fatigue and health-related QoL between mothers of children with ASD and mothers of children without ASD in a Turkish population and investigated the relationship between different aspects of maternal fatigue, depression, and maternal health-related QoL. To the best of our knowledge, this is the first study to have comprehensively investigated the relationship between fatigue and QoL in Turkish mothers of children with ASD and performed multifactorial analysis. The findings of this study confirmed that compared to mothers of typically developing children, the mothers of children with ASD experienced a greater impact of fatigue on mental, physical, and psychological functions. In addition, a higher rate of depressive symptoms and decreased health-related QoL were determined to be strongly linked to high levels of maternal fatigue.

### Differences in Fatigue, Depression, and Quality of Life Between the Mothers of Children and Adolescents with and without ASD

Parents of children with special needs often experience fatigue on both a physical and an emotional level (Giallo et al., 2013). A few studies have investigated the fatigue of mothers of children with ASD in Australia (Giallo et al., 2013; Seymour et al., 2013). Smith et al. (Smith et al., 2010) demonstrated that mothers of children with ASD experienced twice as many fatigue days over the course of 8 days, and Giallo (2013) found that mothers with ASD were 12.17% more fatigued than healthy individuals. In the current study, based on the FSS findings, the mothers of children with ASD were 11.65% more fatigued than the mothers of typically developing children. There was no statistically significant difference between the two groups of mothers, which was most probably due to the structure of the FSS. Although the FSS is a well-known and widely-used unidimensional tool for evaluating fatigue (Whitehead, 2009), the impact of fatigue on functionality is evaluated more frequently than symptom severity (Whitehead, 2009). However, the findings of the current study revealed that fatigue affects the cognitive, physical, and psychosocial functions of the mothers of children with ASD two to three times more than the mothers of typically developing children with moderate to high effect sizes. In addition, the FIS findings showed that mothers of children with ASD experienced 151% more fatigue than mothers of typically developing children with a high effect size. Raising a child with ASD is challenging, especially for mothers living in developing countries, due to a lack of social support, poor nutrition, increased problematic behaviors of the child, insufficient physical exercise, inadequate sleep duration, and financial challenges (Daniels et al., 2017; Giallo et al., 2013; Tekinarslan, 2013; Vasilopoulou & Nisbet, 2016). Fatigue is a distressing subjective feeling that is accompanied by falling levels of both physical and mental energy (Finsterer & Mahjoub, 2014). These factors may contribute to the severe exhaustion experienced by mothers of children with ASD.

Parents of children with ASD have a higher incidence of diagnosed depression and anxiety disorders compared to the general population (Schnabel et al., 2020). In a recent study, Kütük et al. (2021) found that Turkish parents of children with ASD had higher levels of depression and emotional burnout compared to parents of typically developing children. Consistent with the literature, the findings of the current study showed that the mothers of children with ASD had depression scores 124% higher than those of the mothers of typically developing children with a high effect size. This is an expected situation, as the diagnosis of ASD is a heart-breaking blow to parents, and the social, behavioral, and emotional challenges of children with ASD can cause their parents extreme, persistent sadness (Hoefman et al., 2014).

Being a mother to a child with ASD is associated with a greater burden of providing childcare and a low health-related QoL (Kuru & Piyal, 2018; Özgür et al., 2018; Turnage & Conner, 2022). Previous research has shown that mothers of children with ASD had a 7% reduced level of general health, assessed with the Short Form 36 health survey questionnaire (SF-36) than mothers of typically developing children (Wang et al., 2018). Moreover, the SF-36 results demonstrated that the mothers of children with ASD experienced a 6.5% increase in bodily pain, a 12.5% decrease in emotional role, a 15.5% decrease in social functioning, a 5% decrease in physical functioning, an 8% decrease in vitality, and a 16% decrease in mental health (Wang et al., 2018). According to the NHP results of the current study, it was highly striking that mothers of children with ASD reported a 163% increase in pain, a 178% increase in emotional reactions, a 284% increase in social isolation, an 82% decrease in sleep quality, a 90% decrease in physical mobility, and a 65% decrease in energy level than mothers of children with typical development. Based on these data from the NHP, social isolation appears to be the most impacted domain of QoL. These findings in the current study are consistent with the outcomes reported by Daniels (2017), which indicated that parents of children with ASD in Turkey experience stigma (Daniels et al., 2017). The possible explanation for this is the community’s lack of acceptance of autistic behaviors due to inadequate social support (Zaidman-Zait et al., 2017). Therefore, Turkish mothers of children with ASD may prefer to distance themselves from a social environment.

### Relationships Between Maternal Fatigue and Quality of Life

Fatigue is the forgotten aspect of QoL (Seymour et al., 2013). To the best of our knowledge, this is the first study to have investigated the relationship between fatigue and QoL in Turkish mothers of children with ASD using a multifactorial approach and an autism-specific QoL questionnaire for Turkish mothers. The study results showed that the findings of QoLA-Part A, indicating the mothers’ overall perception of QoL, had the most important impact on maternal fatigue. However, the relationship between fatigue and QoLA-Part B, assessing the mothers’ perception of how much of a problem their child’s ASD-related behaviors are, was not found to be statistically significant in the multivariate analysis model. The possible explanation for this could be cultural differences. Using the QoLA questionnaire, Eapen (2023) found that the effects on parents of a child’s ASD-specific difficulties (QoLA-Part B) were highly comparable across countries, and that parents from similar cultural contexts have similar ideas of a child’s ASD-specific behaviors or difficulties (Eapen et al., 2023). In the QoLA-Part B of that study, Turkish mothers claimed that their child’s ASD-related behaviors or challenges had the least impact on their QoL, comparable to the reports of parents of children with ASD in Australia and the United Kingdom (Eapen et al., 2023). Based on the clinical observations for the current study, several Turkish mothers indicated not wanting to respond to specific QoLA-Part B questions, and marked them as “not applicable.”

Fatigue appears to have an impact on cognitive functions, including reduced clarity in thinking, self-attribution of blame, difficulties in decision-making, and impaired memory and concentration (Giallo et al., 2013). The results of this study support the current evidence that a decline in the QoL and physical mobility of mothers, is associated with an increase in the adverse effects of fatigue on cognitive functions. Maternal fatigue has been identified as a contributing factor to difficulties in controlling a child’s behavior, resulting in a worsening of problematic behaviors (Giallo et al., 2013; Seymour et al., 2013). Conversely, there is a correlation between higher levels of maternal fatigue and the emergence of severe problematic behaviors in children with ASD (Seymour et al., 2013). Moreover, mothers tend to use maladaptive coping strategies due to a significant level of fatigue.

The present study results also support the evidence that a sedentary lifestyle is associated with fatigue (Ellingson et al., 2014). According to this research, a reduction in physical mobility and a decrease in a mother’s QoL, in addition to increased social isolation, have a negative effect on the impact of fatigue on physical functions. Mothers with ASD devote a significantly greater amount of time, approximately 40% more, to providing care for their children compared to mothers of typically developing children, and these mothers have fewer opportunities for participating in leisure activities (Smith et al., 2010). Previous studies have shown that depression, anxiety and stress are related to fatigue in the mothers of children with ASD (Giallo et al., 2013; Seymour et al., 2013). Similarly, emotional reactions and the QoL of the child are essential factors that influence the impact of fatigue on psychosocial functions. The possible explanation for this is that the emotional reactions of mothers, such as stress, anxiety, and depressive symptoms, worsen because of the communication and social interaction difficulties, stereotypical behaviors, and limited interests commonly observed in children with ASD (Fombonne et al., 2020).

The primary strength of this study was that the relationship between maternal fatigue and the QoL of mothers was examined using multifactorial analysis and autism-specific QoL questionnaires for parents in a Turkish population. Other strengths of the study were the use of both global and autism-specific QoL questionnaires and the use of valid and reliable assessment tools for Turkish mothers. This study represents a novel investigation into the effects of fatigue on the cognitive, physical, and psychosocial functioning of the mothers of children with ASD, compared to the mothers of children without ASD.

### Limitations and Future Research

However, there were some limitations to this study. The broad spectrum of age groups of the children with ASD may have an impact on maternal fatigue. This is due to the increasing burden of caring for a child with a chronic disorder, which is compounded by additional stressors such as managing medical treatments and therapies, financial demands, and daily caregiving responsibilities. Furthermore, the degree of autism can be evaluated through targeted behavioral assessments. In addition, no qualitative statements from mothers were included which could be considered a limitation in respect of gaining insights into the mothers’ feelings. Future research should focus on the examination of the QoL of children and the associated determinants together with the QoL of mothers and fatigue using face-to-face interviews. Community-based projects and interventions to increase the physical activity of mothers of children with ASD could alleviate fatigue and improve the QoL.

### Implications for Practice

The findings of this study show the importance of fatigue affecting the cognitive, physical, and psychosocial functions of Turkish mothers of children with ASD. The low level of QoL and reduced physical activity are strongly related with an increase in the adverse effects of fatigue on cognitive functions. Therefore, clinicians should focus not only on children with ASD, but also on their mothers. Yoga, meditation, and aerobic exercises should be recommended by rehabilitation team members to improve the physical mobility of mothers of children with ASD. Increasing the mothers’ physical activity can help to reduce fatigue by increasing their vitality and motivation. Group exercise programs in particular may have a stronger positive influence on fatigue and cognitive skills in mothers of children with ASD.

## Conclusion

Fatigue is a significant public health issue in Turkey and globally, significantly reducing QoL for mothers of children with ASD by affecting various aspects of their everyday activities. However, there is limited evidence regarding the relationship between fatigue and QoL in Turkish mothers of children with ASD. The current study has highlighted that the fatigue of Turkish mothers of children with ASD has a great impact on cognitive, physical and psychosocial functions. Furthermore, the QoL, physical mobility, social isolation, and emotional reactions of mothers have a significant impact on maternal fatigue. It was also interesting that the participating mothers were hesitant to discuss that how their child’s ASD-related behaviors were challenging in daily life.
